# Acknowledgement to Reviewers of *Toxins* in 2015

**DOI:** 10.3390/toxins8010031

**Published:** 2016-01-21

**Authors:** Toxins Editorial Office

**Affiliations:** MDPI AG, Klybeckstrasse 64, CH-4057 Basel, Switzerland; toxins@mdpi.com

The editors of *Toxins* would like to express their sincere gratitude to the following reviewers for assessing manuscripts in 2015.

We greatly appreciate the contribution of expert reviewers, which is crucial to the journal’s editorial decision-making process. Several steps have been taken in 2015 to thank and acknowledge reviewers. Good, timely reviews are rewarded with a discount off their next MDPI publication. By creating an account on the submission system, reviewers can access details of their past reviews, see the comments of other reviewers, and download a letter of acknowledgement for their records. In addition, MDPI has launched a collaboration with Publons, a website that seeks to publicly acknowledge reviewers on a per journal basis. This is all done, of course, within the constraints of reviewer confidentiality. Feedback from reviewers shows that most see their task as a voluntary and mostly unseen work in service to the scientific community. We are grateful to our reviewers for the contribution they make.

Aasen, JohnHalwachs, SandraPellett, SabineAbarca, M.l.Hambright, DavidPeña, EstebanAbbas, HamedHammarström, LennartPeng, Chi-chungAbe, TakaakiHan, Sang-baePerrone, GiancarloAbraham, AnnHan, SangMiPerumalsamy, RamarAbraham, DietmarHao, WeilongPestka, JamesAbrunhosa, LuisHardy, MargaretPfister, JimAdam, GerhardHarwood, TimPflugmacher, StephanAdriano, MaroccoHasegawa, MakotoPfohl-Leszkowicz, AnnieAgarwal, ShivangiHassan, Yousef IPiccinini, RenatAh, Chil SeongHatano, TsutomuPicking, WendyAhmed, WarishHayat, AkhtarPietsch, ConstanzeAhn, Chi-yongHedeland, MikaelPillai, Segaran P.Aktories, KlausHendrich, SuzannePinchuk, IrynaAlderman, StephenHendriksen, Peter J.m.Pincus, Seth H.Alfonso, AmparoHENNEKINNE, Jacques- AntoinePineda, Sandy SAli, NurshadHerzig, VolkerPINTON, PhilippeAlmeida, Joana R.Heuck, Alejandro P.Piscia, RobertaAltmann, DMHewlett, Erik L.Pistarini, CaterinaAlvito, Paula CristinaHiggins, Paul J.Pistocchi, RossellaAmadori, M.Hirata, HirokuniPitt, JohnAmano, YoshimasaHo, MengfeiPizzo, FabiolaAmeye, MaartenHodge, David RPlaczek, RichardAmirlak, BardiaHodgson, WaynePodolska, GrazynaAnders, Hans-joachimHolford, MandePollard, PeterAndrade, María J.Holmøy, TrygvePolo, JavierAndreescu, SilvanaHosório, HugoPolo-Parada, LuisAndy Pickett, AndyHostetter, Shannon JPolticelli, FabiatoAnfossi, LauraHovhannisyan, MkrtichPopescu, RuxandraAnniballi, FabrizioHruska, ZuzanaPopoff, Michel R.Antonissen, GuntherHsu, Chih-MingPosthaus, HorstApostolidis, ApostolosHu, Wei-GangPraena, Daniel GutierrezAppelbaum, LiorHuang, Dorothy YuPredel, ReinhardApplegate, ToddHuang, Tur-FuPrescott, John F.Arabatzis, MichaelHuang, Wei-ningProft, MarkusArakawa, ToruHudnell, H. KennethPsarra, Anna-maria G.Arbillaga, LeireHuguet, AntoinePuschner, BirgitArduini, FabianaHumbert, Jean-FrançoisQuiblier, C.ARGILES, AngelHumpf, Hans-UlrichQuinn, JCArias, Renée S.Hunt, Piper ReidQuiñones, BeatrizArimitsu, HideyukiHuq, FazlulQuinton, LoïcAriño, Prof. Dr. AgustínHusmann, MatthiasRagupathy, SubramanyamArmstrong, Glen DHuzella, LouisRajasekaran, KanniahAsai, TeigoHymery, NolwennRalston, EmilyAsgari, SassanIchikawa, NatsukoRamesh, GanesanAsranirk, RKIgbinosa, EtinosaRamos, Vesper Fe Marie LlanezaAsselman, JanaIliev, AsparouhRamsdell, JohnAudenaert, KrisImbriano, CarolRasetti-Escargueil, C.Avelino, AntonioInman, Denise M.Rasmussen, Peter HaveAvrutina, OlgaInoue, KenichiroRasooly, AvrahamAvula, BharathiIntiso, Dott. DomenicoRatajczak, M. Z.Ayalew, SahluIppoliti, RodolfoRatner, AdamB. Ng, TziIsman, Murray B.Reason, DonaldBailly, Jean-DenisJ. FitzGerald, DavidRediske, Richard R.Baines, DanicaJabbari, BahmanReed, KBakke, MereteJaen, Maria TeresaReguera, BeatriceBann, James G.Jagadeesan, RajeswaranReichert, David E.Bargu, SibelJan, Tong RongReichwaldt, ElkeBarr, John R.Jančula, DanielReiss, KarinaBarreau, ChristianJankovic, JosephRekand, T.Basak, Ajit K.Jenkins, CherylResiere, DaborBastiaenen, Caroline H GJenner, RonaldRestivo, Francesco MariaBattacone, GianniJennings, PaulReverberi, MassimoBattillani, PaolaJin, Byung RaeRevilla, PedroBaxa, Dolores V.Johnson, RichardRhee, Jae-SungBayry, JagadeeshJosic, DjuroRicelli, A.Beach, DanielJost, Wolfgang H.Richlen, Mindy L.Becher, Dr. FrançoisJuan, CristinaRichmond, Douglas S.Belanger, Faith C.Juan-García, AnaRiley, RonaldBeloglazova, N.Juillerat‑Jeanneret, LucienneRivas-Perea, PabloBennett, David L. H.Jung, JeeRivera-Posada, JairoBenson, Janet M.Jungo, FlorenceRockey, Daniel D.Benz, RolandJustine R., SchmidtRodicio, María RosarioBerardelli, AlfredoJuttner, FriedrichRodrigues, PaulaBergstrom, Gary C.Kachlicki, PiotrRodriguez de la Vega, RicardoBernardini, ChiaraKahlert, StefanRogers, Michael S.Bernhoft, AkselKalkum, MarkusRohlfs, MarkoBernstein, Kenneth E.Kamm, ChristophRohrlack, ThomasBerrada, HoudaKane, Douglas D.Roncada, PaolaBerthiller, FranzKanematsu, S.Rong-Hwa, ShyuBertram, RalphKarjalainen, MiinaRossi, FilippoBevilacqua, AntonioKarlovsky, PetrRoze, Ludmila V.Beyer, M.Karp, Barbara IllowskyRúa, Megan A.Bills, GeraldKatikou, PanagiotaRubert, JosepBiron, David G.Kaushal, Gur P.Ruiz, InigoBitar, Khalil N.Kawada, ManabuRychlik, MichaelBlachier, FrançoisKawakami, KojiRyu, Ji-WonBlanco, JuanKearney, Christopher M.Sabatier, Jean-MarcBlasi, JuanKeith, FosterSaint-Cyr, Manuel J.Blumenfeld, AndrewKem, William R.San Juan Serrano, FuencislaBoles, Blaise R.Kim, BumseokSánchez Alcázar, JoséBondy, GenevieveKim, EuikyungSantamato, AndreaBonham, Andrew J.Kim, HeonSantos, CledirBorges, HugoKim, Min GonSarrocco, SabrinaBosilevac, MickKim, Sung OukSasaki, TetsuhikoBosmans, FrankKimura, MakotoSaul, NadineBotana, LuisKip, PanterScala, ValeriaBouaïcha, NoureddineKiris , Erkan Schatzmayr, GerdBougis, PierreKiss, Robert S.Schaumburg, FriederBowers, Erin L.Kistler, H. CorbyScheib, HolgerBowling, LeeKita, MasakiSchein, Catherine H.Boyd, Robert S.Kitani, SSchelin, JennyBoyer, Anne E.Klimaschewski, LarsScheutz, FlemmingBradley, Walter G.Klitzke, SondraSchierack, PeterBrandon, DavidKluess, JeannetteSchmale, DavidBraun, VolkmarKnölker, Hans-JoachimSchmidt, GudulaBraunbeck, ThomasKoh, Chung-YanSchmidt-Heydt, MarkusBreckpot, KarineKoizumi, AkioSchwaninger, MarkusBrena, CarloKoizumi, SchuichiSchweiger, WolfgangBresciani, MarianoKojima, TakashiScipinotti, R.Brin, Mitchell F.Kolf-Clauw, MartineScott, BarryBrookes, JustinKomatsu, MasaharuSegura, MatildeBrown, Euan R.Kong, Siu-KaiSeifert, RolandBrown, RobertKonno, KatsuhiroSenerpont Domis, Lisette DeBrunner, KurtKool, JeroenSeo, Jong BokBucciarelli, Gary M.Korotkov, Konstantin V.Serrano, A.B.Budrys, EduardasKorotkova, NataliaServin, Alain L.Buehler, Paul W.Kouadio, James HalbinSesardic, TheaBulaj, GrzegorzKoudelka, GeraldSeto, YasuoBulla, Lee A.Kowalewski, M. P.Shakya, MigunBurtey, StéphaneKrepkiy, Dmitriy VShan, XueyanBusman, MarkKrienitz, LotharSharma, ShashiButrón, AnaKuehn, Meta J.Shaw, LindseyCahill, Patrick L.Kuo, Hann-ChorngShaw, Pang-ChuiCai, ShuoweiKurosawa, ShinichiroShepherd, Suzanne M.Callebaut, AlfonsKwak, Jong-YoungSherman, Michael P.Calleros, LauraKwon, Hyung-JinShimizu, TakeshiCalvete, Juan J.L. Tesh, VernonShiomi, KazuoCalvo, Ana M.Lacy, D. BordenShoemaker, Charles B.Cammarata, MatteoLaganà, AldoShupp, Jeffrey W.Carlotta, BalconiLambeau, GérardSieira, PabloCasadevall, ArturoLampert, AngelikaSiemens, JanCasewell, Nicholas R.Lance, EmilieSignorini, MassimoCaudle, RobertLandini, PaoloSilberstein, StephenCerasino, LeonardoLang, Mark L.Silva, Christopher J.Chae, Han-JungLaw, Betty Yuen-KwanSimone, GiuseppinaChampion, MatthewLawrence, JimSlot, Jason C.Chan, ThomasLawrence, William S.Smith, JulietteChan, WanLean, I.J.Smith, Laura E.Chang, Perng-kuangLeão, Pedro N.Smith, Val H.Chatzaki, EkateriniLebedev, Albert T.Smyth, Joan A.Cheli, FedericaLee, Jean C. Snape, WilliamChen, ChiLee, Si HyeockSoares, AndreimarChen, Jih-JungLee, Tzong-shyuanSolfrizzo, MicheleChen, RongLefebvre, Kathi A.Song, JeongminChen, Shih-ShunLeistner, EckhardSoto, JulioChen, TianbaoLesieur, ClaireSpadaro, DavideCheng, Luisa W.Levy, HaimSpangenberg, BerndChirumbolo, SalvatoreLi, Pei-WuSquires, E.J.Chizzola, RemigiusLiedberg, Bo GunnarStanker, Larry H.Cho, Dong HyungLima, CarlaStegmayr, BerndCho, YukoLimón, M. Carmen Stessl, BeatrixChoi, Sank HoLimonciel, AliceStiborova, MarieChristine, BeetonLin, GlennStiles, Bradley G.Christos, GeorgiouLingueglia, EricStöcklin, RetoChui, LindaLingwood, Clifford AStoker, Tammy E.Cianchetti, CarloLippolis, VincenzoStournaras, ChristosCinar, Hediye NeseLisacek, FrederiqueSturm, Gunter J.Citores, Lucia Liu, Biing-HuiSubramaniam, RajagopalCiufolini, Marco A.Liu, Shing-HwaSuga, H.Cohen, GeraldLivrea, Maria AntoniaSugai, MotoyukiCollett, Mark G.Lopot, FrantišekSugita-Konishi, YohshikoComella, Cynthia L.Louzao, M. CarmenSugiyama, MasanoriCompagnone, DarioLuo, MengSulyok, MichaelComte, KatiaLürling, MiquelSuman, Devi S.Corbett, Cindi R.Luvisetto, SiroSun, Der-ShanCosta, Pedro ReisMace, Wade J.Sun, HongCote, ChristopherMaeda, KazuyaSun, Shu-FenCrane, John K.Magnus, KarlssonSunagar, KartikCross, Alan S.Maicas, SergiSuntres, ZachariasCroubels, SiskaMañes, JordiSwartz, KentonCruz, CeliaManning, JaneSwearengen, JamesCruz, FranciscoMaragos, ChrisSwevers, LucCummings, BrianMarceau, FrançoisSzabados, FlorianCytlak, UrszulaMarcet-Houben, MarinaTaira, JunseiCzabotar, Peter E.Maresca, MarcTakahashi, K.G.Dai, Susie Y.Mari, FrankTakeda, SoichiDamann, Kenneth E.Marie, BenjaminTallent, Sandra M.Dänicke, SvenMarín, SoniaTanaka, EijiDanneels, Ellen L.Marino, AngelaTanaka, HiroyukiDarós, José-AntonioMariottini, GianluigiTanaka, ToshiharuDavenport, AnthonyMarte, AntonioTanaka, YoshikazuDavid, HelenaMartelletti, PaoloTanimoto, HirokiDavis, Timothy W.Marzocco, StefaniaTardieu, DidierDavletov, BazbekMasereeuw, RosalindeTaylor, BruceDayeh, Vivian R.Mathieu, FlorenceTerzi, ValeriaDe Baere, S.Matsuda, Tomonari Tester, PatriciaDe Boer, Hugo J.Matsuka, YoshizoThibodeau, JacquesDe Boevre, MartheMattei, CesarThorpe, ChelesteDe Curtis, FilippoMatumba, LimbikaniThrane, Ulfde Haro, LucMatysiak, JanThyagarajan, BaskaranDe Hertogh, WillemMaul, RonaldTittlemier, Sheryl A.De La Cruz, Armah A.Maupin, Owen M.Toebe, KerstinDe Mil, ThomasMaynard, Jennifer A.Tolosa, JosefaDe Sarro, GiovambattistaMayne, ChristopherTorres-Russotto, DiegoDebRoy, ChitritaMazora, OhadTortorella, DomenicoDees, W. LesMcCarron, PearseTouchard, AxelDegen, Gisela H.McClain, Mark S.Tournier, Jean NicolasD'Elios, MarioMcClane, BruceTrachtman, HowardDemirev, PlamenMcCormick, JohnTrematerra, PasqualeDenny, Paul W.McCormick, SusanTrim, Steven A.DeRosa, Maria C.McFarland, KatherineTrischitta, FrancescaDevenish, RodMckillip, John L.Trojanowicz, BoguszDevreese, MathiasMcLachlan, CraigTruong, DanielDevriendt, BertMcmorrow, TaraTsuge, HideakiDi Maro, AntimoMcNabb, PaulTucker, StevenDi, RongMeggs, William J.Tudzynski, BettinaDias, ElsaMelnyk, Roman A.Tudzynski, PaulDmochowski, RogerMelton-Celsa, AngelaTufféry, PierreDolara, PieroMerino, GraciaTumer, NilgunDomashevskiy, Artem V.Merlot, ElodieTurk, TomDorantes-Aranda, Juan JoséMerrick, B. AlexTurnbull, W. BruceDorner, Brigitte G.Mesquita, Francisco SarmentoTurner, PaulDosio, FrancoMetcalf, Jordan P.Tzang, Bor-ShowDossena, SilviaMetzler, ManfredUchiumi, ToshioDotsika, EleniMeulenberg, ElineUeda, KazumitsuDou, LaetitiaMicheli, Laura Umemura, MycoD'Ovidio, RenatoMiddleton, John R.Undheim, EivindDowell, Floyd E.Miller, DavidUndheim, Eivind, A.B.Duarte, SofiaMirasoli, MaraVago, RiccardoDubey, Mukesh K.Miyashita, MasahiroValentovic, MonicaDuca, RaduMobli, MehdiValério, ElisabeteDuringer, Jennifer M.Mogridge, JeremyValls, MarcDuso, CarloMohamadzadeh, MansourVan den Top, HesterDzantiev, BorisMolgo, JordiVan der Fels-Klerx, H.J.Eichacker, Peter Q.Montgomery, JennyVan der Merwe, DeonEirín-López, José M.Moon, YuseokVan Dolah, FrancesEkuni, DaisukeMoore, GeromyVan Eijk, MarcoEleftheriadis, TheodorosMoran, YehuVan Klink, JohnEl-Nezami, Hani SaidMorelli, Adrian E.Van Leeuwen, Hans C.El-Shehaw, RehabMorita, KyojiVan Pamel, ElsEmelko, Monica B.Morse, StephenVan Wijk, RichardErba, Elisabetta BoeriMosiello, GiovanniVanhaecke, LynnEriksson, JohanMrsny, Randall J.Vanheule, AdriaanEscames, GermaineMuench, StephenVarga, ElisabethEscriche, BaltasarMüller, Marina E.H.Vasconcelos, VitorEtxebeste, OierMüller, UlrichVázquez-de-Aldana, Beatriz R.Evans, SusanMulley, John F.Velliyagounder, KabilanEven, SergineMunday, RexVelotta, RaffaeleFagerquist, Clifton K.Muñoz-Guerra, Mario FernandoVenancio, ArmandoFaggio, CatarinaMurakami, MakotoVenier, PaolaFalconer, IanMurch, SimonVerbrugghe, ElinFalconer, Ian R.Müthing, JohannesVetter, IrinaFanelli, FrancescaNabok, AlexeiVetvicka, VaclavFarrell, HazelNagahama, MasahiroVidal, NicolasFattal-Valevski, AvivaNagashima, HitoshiViegas, SusanaFeng, Peter.C.H.Nagashima, YujiVierra, CraigFernandes-Ferreira, ManuelNagata, KojiVignes, MichelFerrari, LucaNaidoo, NirinjiniVodyanoy, VitalyFerrer, Emilia Nakane, AkioVon Eichel-Streiber, ChristophFerreras, JosÉ MiguelNanjundan, M.Von Tiedemann, AndreasFibach, EitanNathanail, Alexis V.Voss, Kenneth A.Figadere, BrunoNaumann, MarkusWalker, AndrewFinley, Natosha L.Negishi, TomoeWalker, Mark J.Flewelling, LeanneNeiman, MWard, AnthonyFox, Edward MNelsen, David R.Warth, BenediktFox, JayNestorovich, EkaterinaWaskiewicz, AgnueszkaFrantisek, MalirNet, SopheakWassmer, ThomasFranz, EelcoNewton, RobertWatanabe, RyuichiFrech, MoritzNg, Kenneth K. S.Weaver, MarkFriesema, IngridNg, Tzi BunWei, LipingFrisvad, Jens ChristianNicholson, PaulWeiss, John H.Fritsche, JanNie, DaotaiWeller, Michael G.Frucht, David M.Niehuis, OliverWerren, John H.Fusco, VincenzinaNijnik, AnastasiaWertz, PhilipGallo, AntonioNishida, YoshihiroWhite, James FGamazo, CarlosNoguchi, TamaoWhittington, Camilla M.Garber, Eric A.E.Norton, RaymondWilcox, ChristieGarcía-Caballero, MelissaNossol, ConstanzeWilderling, WillemGard, PaulNowrouzian, Forough L.Wilkenfeld, Ari Jacob LeviGarner, BiancaO’Donnell , Michael J Williams, PaulGazerani, ParisaObermair, Gerald J.Williamson, E DianeGeisen, RolfOda, MasatakaWilson, Brenda A.Gentile, SaverioOikawa, HideakiWindham, GaryGerhard, RalfO'Kennedy, RichardWinkel, Kenneth D.Geue, LutzO'Kiely, PadraigWolz, ChristianeGhadouani, AnasOkumura, ShiroWong, Jon W.Giannantoni, AntonellaOppert, BrendaWood, SusannaGilbert, Robert J.C.Orlov, Sergei N.Wright, Jeremy J.Gilles, NicolasOrth, Joachim H. C.Wright, MaureenGilly, W.F.Osborne, JamesWu, Chin-ChungGilmartin, NiamhOscherwitz, JonWulff, HeikeGiovannoli, CristinaOssiprandi, Maria CristinaWürzner, ReinhardGiuberti, GianlucaOswald, IsabelleXu, PeishengGnonlonfin, G. J. B.Ota, MasaoYahiro, KinnosukeGoldmana, Ellen R.Otero, PazYaksh, TonyGomelsky, AlexOtten, TimothyYamada, KeikoGong, Yun YunOvenden, SimonYamashita, Mari YotsuGonzález-Maeso, JavierOvsepian, SaakYamazaki, MasahitoGordon, Donna M.Özbek, SuatYeager, Linsey A.Goto, ShunsukePagliuca, GiampieroYin, Chang-SikGoulding, Celia W.Palma, LeopoldoYin, GuohuaGovind, ShubhaPalmer, MikeYin, Mei-ChinGovindarajan, RajagopalanPalumbo, Jeffrey D.Yli-Mattila, TapaniGower, JimPanaccione, DanielYoon, Moon-YoungGraham, H. KerrPanaccione, Daniel G.Yoshinari, TomoyaGrazzi, LiciaPanagou, EfstathiosYoung, CarolynGreen, BenPando, ConcepciónYu, Feng-yihGreenfield, Dianne I.Papatsoris, Athanasios GYunus, AghaGressent, FrédéricPappas, AthanasiosZamyadi, ArashGrilli, EsterParamithiotis, SpirosZanotti, GiuseppeGruber, ChristianParente, AugustoZaslawski, ChrisGuerre, PhilippePark, Hyun-WooZebeli, QendrimGuo, BaozhuPark, Jong-HyunZeleny, ReinhardGurevich, Eugenia V. Park, Kwan-KyuZendong, ZitaGutierrez-Nibeyro, Santiago D.Pascale, MichelangeloZentek, JürgenGuzmán-Guillén, R.Pascual, JulioZhang, BaolinGwenin, ChristopherPascual-Ahuir, AmparoZhang, KaiHadjipanayis, Costas G.Pasetti, Marcela F.Zhou, TingHagenacker, TimPatel, Abhijit A.Zilberberg, NoamHahm, Ki BaikPaterson, R Russell MZobel-Thropp, Pamela A.Hahm, Young TaePattono, D.Zoja, CarlaHait, JenniferPayan-Carreira, Rita
Hallegraeff, GustaafPeigneur, Steve
Hallen-Adams, Heather

Hallock, Robert M.



